# Molecular mechanisms of retinal ganglion cell degeneration in glaucoma and future prospects for cell body and axonal protection

**DOI:** 10.3389/fncel.2012.00060

**Published:** 2013-01-09

**Authors:** Yasunari Munemasa, Yasushi Kitaoka

**Affiliations:** Department of Ophthalmology, St. Marianna University School of MedicineKawasaki, Kanagawa, Japan

**Keywords:** glaucoma, optic nerve, retinal ganglion cell, axonal degeneration, axonal protection

## Abstract

Glaucoma, which affects more than 70 million people worldwide, is a heterogeneous group of disorders with a resultant common denominator; optic neuropathy, eventually leading to irreversible blindness. The clinical manifestations of primary open-angle glaucoma (POAG), the most common subtype of glaucoma, include excavation of the optic disc and progressive loss of visual field. Axonal degeneration of retinal ganglion cells (RGCs) and apoptotic death of their cell bodies are observed in glaucoma, in which the reduction of intraocular pressure (IOP) is known to slow progression of the disease. A pattern of localized retinal nerve fiber layer (RNFL) defects in glaucoma patients indicates that axonal degeneration may precede RGC body death in this condition. The mechanisms of degeneration of neuronal cell bodies and their axons may differ. In this review, we addressed the molecular mechanisms of cell body death and axonal degeneration in glaucoma and proposed axonal protection in addition to cell body protection. The concept of axonal protection may become a new therapeutic strategy to prevent further axonal degeneration or revive dying axons in patients with preperimetric glaucoma. Further study will be needed to clarify whether the combination therapy of axonal protection and cell body protection will have greater protective effects in early or progressive glaucomatous optic neuropathy (GON).

## Introduction

The axons of human retinal ganglion cells (RGCs) are approximately 50 mm in length and form synapses with cells in the lateral geniculate nucleus of the thalamus. The axons are arranged in bundles separated and ensheathed by glial cells. Upon exiting through the lamina area, the axons become myelinated with oligodendrocytes. Glaucomatous optic neuropathy (GON), the second leading cause of blindness worldwide, is a neurodegenerative disease characterized by structural damage to the optic nerve and the slow progressive death of RGCs (Quigley, 1996; Resnikoff et al., 2004). Previous reports indicated that GON initially occurs in the lamina area and is associated with several factors, such as disruption of neurotrophic factor, glial activation, and release of tumor necrosis factor (TNF), oxidative stress, dysregulation of the immune system, and mitochondrial dysfunction (Quigley and Addicks, 1980; Tezel and Wax, 2000; Schori et al., 2001, Tezel, 2006; Ju et al., 2009). These changes contribute to deformation of the lamina and subsequently activate several molecular pathways to induce axonal degeneration and RGC body death. Therefore, an understanding of the mechanism of axonal degeneration is needed to develop new strategies for glaucoma treatment.

RGC bodies, which carry the final neuronal output of the retina, receive visual signals from photoreceptors via the two preceding layers of neuronal cells, bipolar and amacrine cells, and transmit that information to the brain. Organelles of RGCs, including the mitochondria, endoplasmic reticulum (ER), Golgi, and cytoplasm, move from their sites of biogenesis in the cell body to distant axons and maintain the cellular environment. The cell body controls cellular organelles, and therefore cell body death influences their movements and functions (DePina and Langford, 1999). Apoptosis is a major category of cell body death signaling, and its characteristics are nuclear fragmentation, chromatin condensation, DNA fragmentation, and cell shrinkage (Penfold and Provis, 1986; Smith et al., 1989). Although most cell body death occurs following axonal degeneration, the immune system, glial activation, and oxidative stress also contribute to cell body death in the retina independently axonal degeneration. Consequently, the mechanism of cell body death due to GON must also be elucidated.

The reduction of intraocular pressure (IOP) remains the main strategy to slow the progression of glaucoma. However, thinning of neurofibers and progression of visual field defects are observed even in eyes in which IOP is reduced, suggesting that disease progression is at least partially independent of IOP, especially in normal tension glaucoma (Harbin et al., 1976; Caprioli, 1997; Georgopoulos et al., 1997; Leske et al., 2003). Therefore, the establishment of a neuroprotective strategy in GON is necessary. Although many papers focusing on cell body protection have been published recently, there are few neuroprotective agents for clinical application. Since the primary site of GON is the optic nerve in the lamina, axonal protection has become an essential strategy for neuroprotective interventions, including secondary cell body death.

In this review, we describe the molecular mechanisms of axonal degeneration and cell body death in GON and discuss the future prospects for neuroprotection.

## Cell body injury

### IOP elevation and RGC death

Glaucoma is complex, multifactorial disease characterized by axonal degeneration and RGC loss. The non-human primate optic nerve shows close anatomic similarities to the human one, with multiple layers of collagen and elastic tissue in the lamina cribrosa (Anderson, 1969, 1970). While the non-primate, such as rat and mouse, optic nerve head lacks that specific similarity to the primate one, several other similarities are seen. Instead of the lamina cribrosa, a lamina beam or glial lamina, of which the astrocyte processes are in intimate contact with axons, occurs in the lamina area (Morrison et al., 1995). Experimental primate glaucoma indicates that IOP elevation itself may induce specific changes in the composition of the optic nerve head extracellular matrix (ECM) and obstruction by the lamina cribrosa in otherwise normal eyes, including altered collagen and elastin fibrils and deposition of several macromolecules within the pores, leading to axonal degeneration and subsequent RGC death (Morrison et al., 1990; Quigley et al., 1991a,b). In contrast, non-primate experimental glaucoma models induces axonal degeneration without banding of the lamina cribrosa, indicating that molecular changes in the optic nerve are also essential factors in axonal degeneration and RGC death.

RGC body death induced by high IOP also results in secondary degeneration through several molecular changes. Interruption of the retrograde axonal transport of neurotrophic factors has been postulated as a mechanism of secondary RGC degeneration (Quigley and Addicks, 1980). Furthermore, disruption of the expression of the axonal motor protein dynein is observed in the optic nerve head. Therefore, modulation of axonal transport is a crucial event in GON (Martin et al., 2006).

IOP elevation induces oxidative stress in RGCs through decreased activity of several enzymes comprising the antioxidant defense system, including superoxide dismutase (SOD), glutathione peroxidase, and catalase, and has been implicated in RGC body death (Moreno et al., 2004). Furthermore, we previously found that decreased thioredoxin 1 (Trx1) and thioredoxin 2 (Trx2) levels, which play critical roles in the regulation of oxidative stress, are observed in the glaucomatous retina, and overexpression of these proteins supports RGC survival (Munemasa et al., 2008).

### Oxidative stress in the retina

Oxidative stress occurs when the generation of reactive oxygen species (ROS) exceeds the cellular ability to neutralize and eliminate them. ROS have been implicated as crucial factors in neurodegenerative diseases, such as Alzheimer, Parkinson, and Huntington diseases (Di Matteo and Esposito, 2003; Barber et al., 2006; Browne and Beal, 2006; Savitt et al., 2006). ROS are generated predominantly in mitochondria through a variety of processes such as normal aerobic metabolism and second messengers in various signal transduction pathways. H_2_O_2_ is proposed as a major contributor to oxidative damage by ROS after conversion from superoxide released from mitochondria. Once generated, H_2_O_2_ may further impair mitochondrial electron transport and enhance ROS production. When H_2_O_2_ is present at high or sustained levels, it induces severe damage not only to mitochondria but also to cellular proteins, lipids, and nucleic acids, and subsequently causes cell body death.

Retinal injury due to oxidative stress has been reported in various animal models, including axotomy, ischemia-reperfusion, *N*-methyl-D-aspartate (NMDA), and experimental glaucoma (Kikuchi et al., 2000; Atlante et al., 2001; Tezel, 2006). Optic nerve axotomy causes ROS production through glial activation, disruption of trophic factors, and subsequent RGC death. Our previous study showed that axotomy of the optic nerve causes a change in redox status, indicating an increase in Trx1 and Trx2 levels in isolated RGCs. Overexpression of Trx1 and Trx2 prevents axotomy-induced RGC loss, indicating the critical role of the redox status in axotomized RGC degeneration (Munemasa et al., 2006).

Tissue hypoxia is also associated with RGC degeneration through oxidative injury. Hypoxia-inducible factor-1 alpha (HIF-1α), an O_2_-regulated transcriptional activator, has been identified as a key modulator of O_2_ homeostasis. Upregulation of HIF-1α mRNA is observed in RGC-5 cells under H_2_O_2_-induced oxidative stress (Chen et al., 2012). HIF-1α under hypoxia induces the expression of various transcription factors including those encoding erythropoietin, glucose transporters, glycolytic enzymes, vascular endothelial growth factor, inducible nitric oxide synthase (NOS), and heme oxygenase 1, and their protein products increase the delivery of or facilitate metabolic adaptation to hypoxia (Wang et al., 1995; Iyer et al., 1998). The early increase in the expression of HIF-1α is accompanied by prosurvival factors, such as heat-shock protein (Hsp)27 and 72. On the contrary, proapoptotic molecules including p53 and caspase are observed in the retina under long-term hypoxic conditioning (Tezel and Wax, 1999; Wax et al., 2008). Thus, while the early increase in HIF-1α is accompanied by the activation of adaptive prosurvival factors, long-term exposure to hypoxia induces the activation of proapoptotic signaling. The expression of HIF-1α during RGC degeneration is more evident in human glaucomatous donor eyes, supporting the occurrence of sustained tissue hypoxia in glaucoma. HIF-1α immunoreacitivity is observed not only in the optic nerve but also in the retina in glaucoma (Tezel and Wax, 2004). These observations indicate that hypoxic stress in glaucoma is the initial stress, followed by secondary degeneration.

It has been reported that RGCs are vulnerable to oxidative stress, especially to SOD1 knockout (Yuki et al., 2011). An increase in ROS is observed in the RGCs of SOD1-deficient mice. Furthermore, RGC degeneration precedes the degeneration of other layers in SOD1-deficient mice, indicating the greater vulnerability of RGCs to oxidative stress compared with other neurons. RGC death following axotomy of the optic nerve is due to the bursting of RGC bodies by intracellular superoxide (Kanamori et al., 2010). ROS accumulation in response to axotomy of the optic nerve induces RGC degeneration through changes in the mitochondrial electron transport chain (Lieven et al., 2006). Mitochondrial dysfunction has been also reported in RGC body death in oxidative stress. TNF and buthionine sulfoximine (BSO), an inducer of oxidative stress, cause increased mitochondrial membrane permeability, with a concomitant decrease in Trx2 levels, and subsequently translocation of the apoptosis-inducing factor (AIF) from mitochondrial inner membrane space to the nuclei of RGC-5 cells. Inhibition of mitochondrial permeability by Trx2 overexpression prevents the cytotoxic effects induced by oxidative stress (Munemasa et al., 2009). Thus, mitochondria are one of the target organelles of oxidative injury, and modulation of mitochondria may become a strategy to reduce oxidative stress.

### Autoimmune regulation and RGC death

Ocular immune privilege regulates immune responses, thereby controlling potentially damaging and sight-threatening autoimmune disease (Gregerson, 1998; Streilein et al., 2000). In the central nervous system (CNS), activated T cells were shown to migrate to the site of injury, suggesting that homing T cells that encounter their related antigens at the lesion site and contribute to lesion repair. Apoptotic elimination of T cells is an essential protective mechanism to prevent inflammation and antigen contact in the eye through naturally occurring CD4^+^ CD25^+^ regulatory T cells (Treg) (Peitsch et al., 1993; Kipnis et al., 2002). CD4^+^ activation requires an interaction between the antigen-restricted T cell receptor of the T cell and major histocompatibility complex class 2, an antigen present on the surface of antigen-presenting cells, such as B cells, macrophages, microglia, and dendritic cells (Bretscher, 1992). The accumulation of T cells that migrate to injured tissue likely contributes to the endogenous protective mechanism, but their activity is not sufficient to have a perceptible effect. Therefore, it may be necessary to increase the number of migrating T cells to prevent neurodegeneration. Regulation of autoimmune T cells reactive to myelin antigens can preserve the organism following neuronal injuries (Hauben et al., 2000). Furthermore, T cells specific for copaxone 1, a compound used in the treatment of multiple sclerosis, inhibit RGC loss after optic nerve crush (Kipnis et al., 2002). However, once T cells are presented with myelin antigen, they can also initiate an immune response leading to neurodegeneration. Therefore, modulation of the autoimmune response may be important to develop a new strategy for neuroprotection.

### Glial cell activation and RGC death

Glial cell activation occurs in neurodegeneration through various molecular changes. Gliosis is characterized as the proliferation and hypertrophy of glial cells and results in upregulation of intermediate filaments, such as glial fibrillary acidic protein (GFAP), vimentine, and nestin, and thickening of large cells. Upregulation of the intermediate filaments places them in contact with cytoskeletal components and the ECM, enabling them to initiate rapid cytoskeletal remodeling and exert long-term effects on tissue structure. In the retina, there is an additional glial cell type, the Müller cell, which is a specialized radial glial cell that spans the entire thickness of the retina (Laties and Nichols, 1971) and connects all retinal neuronal somata and processes. In the healthy retina, Müller cells contribute to maintaining the retinal structure and functions, such as glucose metabolism (Poitry-Yamate et al., 1995), ion maintenance (Tsacopoulos and Magistretti, 1996), regulation of retinal blood flow (Paulson and Newman, 1987), uptake and recycling of neurotransmitters (Matsui et al., 1999), and release of various factors. Although retinal neurons are highly susceptible to various forms of injury including ischemia and high IOP, Müller cells are strikingly resistant to stress. Müller cell gliosis in response to various stresses, such as ischemia-reperfusion (Pannicke et al., 2005), axotomy of the optic nerve (Chun et al., 2000), excessive glutamate (Honjo et al., 2000; Kawasaki et al., 2000), and ocular hypertension (Carter-Dawson et al., 1998), has an endogenous protective function. The mechanism by which acute activation of Müller cells is neuroprotective after injury is due to the release of neurotrophic factors, such as brain-derived neurotrophic factor (BDNF), ciliary-derived neurotrophic factor, and pigment epithelium-derived factor, and their antioxidant function (Ju et al., 1999; Oku et al., 2002; Sato et al., 2008; Unterlauft et al., 2012). Conversely, chronic activation of the Müller glia is recognized as an indicator of ongoing neuroinflammation in retinal neurodegeneration. TNF-α and interleukin (IL)-1β, which are major cytokines produced by activated glial cells, are linked to inflammatory processes and mediate RGC death through several pathways, including nerve factor (NF)-κB activation, autophagy regulation, nitric oxide (NO) synthesis, and inflammasome assembly (Tezel and Wax, 2000; Kitaoka et al., 2007; Tezel et al., 2012). Furthermore, NOS 2, which is expressed in the presence of cytokines, is synthesized in activated Müller cells. NO produced in high concentrations by the inducible isoform expressed in activated Müller cells can be neurotoxic to the cells. Thus, activated Müller cells have dual functions depending on the time course after injury.

When exposed to inflammatory stimuli, RGCs enter an active regenerative state, which helps their neurons to survive injury and regenerate lengthy axons through the injured optic nerve (Fischer et al., 2001; Yin et al., 2003). Intravitreal injection of Pam3 Cys, a TLR2 agonist, induces the upregulation of ciliary neurotrophic factor and GFAP expression in the glia, accompanied by the activation of the JAK/STAT3 pathway in RGCs. Consequently, RGCs are converted to the regenerative state, indicated by significant upregulation of GAP43 expression and increased neurite outgrowth of RGCs in culture (Hauk et al., 2010).

Müller cells undergo a proliferative response after neurotoxic injury by re-entering the cell cycle. Many of these cells begin to lose their Müller glial phenotype and express a neurofilament transcription factor common to retinal progenitor cells, such as Pax6, Chx10, and CASH-1, indicating Müller cell dedifferentiation into retinal progenitors and subsequent formation of new retinal neurons (Fischer and Reh, 2001). These observations suggest that Müller cells may play a role in neural regeneration in response to neural injury.

### ER stress and RGC injury

The ER is an important subcellular organelle responsible for the proper folding and sorting of proteins. Only properly folded proteins can be transported to the Golgi body for further processing. In addition, the ER also serves as a dynamic pool of Ca^2+^, governing intracellular Ca^2+^ homeostasis (Görlach et al., 2006). It is susceptible to various stresses that provoke the accumulation of unfolded proteins in the ER lumen. Three ER-related proteins, inositol-requiring enzyme-1, activating transcription factor 6, and protein kinase RNA (PKR)-like ER kinase (PERK), are involved in the initial signaling to the cell through the unfolded protein response.

Previous studies found that increased ER stress in response to ischemia-reperfusion, tunicamycin, the commom ER stress inducer NMDA, and IOP elevation induces apoptotic RGC degeneration accompanied by increased ER stress-related proteins, such as Bip, PERK, and CHOP (Shimazawa et al., 2007; Inokuchi et al., 2009; Doh et al., 2010). Intravitreal injection of tunicamycin results in RGC loss and reduced thickness of the inner retina. Pharmaceutical induction of Bip, an ER chaperone that facilitates protein folding and reduces ER stress, attenuates the retinal expression of CHOP and activation of the apoptotic cascade and inhibits apoptotic RGC death (Inokuchi et al., 2009). Furthermore, CHOP knockout mice show resistance to NMDA-induced retinal injury (Awai et al., 2006). Thus, ER stress plays a pivotal role in retinal neuronal degeneration, and modulation of its activity may become a new strategy for neuroprotection against retinal injury.

### Excitotoxicity

Glutamate, a major excitatory neurotransmitter, is implicated in several ocular diseases, including optic neuropathy and diabetic retinopathy. Glutamate receptors are classified as metabotropic and ionotropic types, the latter of which are further subcategorized into NMDA, α-amino-3-hydroxy-5-methyl-4-isoxazolepropionic acid (AMPA) receptors, and kainate receptors. These receptors are encoded by at least six gene families: a single family for AMPA receptors (GluR1, 2, 3, 4); two for kinate (GluR5, 6, 7 and KA 1, 2); and three for NMDA (NR1, NR2A, B, C, D, and NR3A, B) (Dingledine et al., 1999). Glutamate neurotoxicity has been predominantly linked to excessive stimulation of NMDA receptors, which are activated by coagonist NMDA (glutamate) and glycine. Stimulation of NMDA receptors is observed in the retina in various animal models of neurodegeneration, such as experimental glaucoma, axotomy, ischemia-reperfusion, and NMDA injection (Lam et al., 1997, 1999; Kikuchi et al., 2000; Dong et al., 2008). Intravitreal injection of NMDA causes relatively acute neuronal death, especially in the inner retina, through several molecular pathways. The increase in intracellular Ca^2+^ influx is the initial key molecular event in NMDA receptor-mediated cell death (Laabich et al., 2001). Intracellular Ca^2+^ overload activates Ca^2+^-dependent enzyme systems such as calpain and calcium/calmodulin-dependent kinase 2 (CaMK) (Chiu et al., 2005; Takeda et al., 2007). Calpain is localized in TUNEL-positive apoptotic cells in the inner retina after NMDA injection, and inhibition of calpain resulted in less NMDA-induced neuronal cell death, suggesting a proapoptotic role of calpain in NMDA-induced neutrotoxicity. In contrast, phosphorylation of CaMK2 is observed in the retina relatively early after NMDA injection, and inhibition of CaMK2 synthesis accelerates NMDA-induced RGC loss, indicating an antiapoptotic role of CaMK2 (Takeda et al., 2007). Intracellular Ca^2+^ influx also affects mitochondrial activity, such as the release of cytochrome C and ROS, and results in the activation of several apoptotic pathways. Our previous studies on the downstream intracellular Ca^2+^ influx led us to propose that the activation of proapoptotic molecules, such as NF-κB p65 and p38, c-Jun *N*-terminal kinase (JNK), and c-Jun, plays a role in NMDA-induced neurotoxicity (Kitaoka et al., 2004; Munemasa et al., 2005, 2006; Takada et al., 2011). An inflammatory response, i.e., the upregulation of IL-1β, is also observed in the glia and RGCs after NMDA administration, suggesting the involvement of inflammation in response to excitotoxicity. Thus, although NMDA penetrates into the inner retina after intravitreal injection and induces various molecular changes in the glia and RGCs which lead to RGC apoptotic degeneration and inner retinal thinning, those changes may also affect optic nerve degeneration. Our previous study showed that axonal degeneration with neurofilament loss is evident 3 days after intravitreal injection of NMDA (Kuribayashi et al., 2010). These changes occur after TUNEL-positive DNA fragmentation in RGCs, indicating anterograde degenerative change in NMDA-induced neurotoxicity. Furthermore, apoptotic cell body death affects axonal transport to axons through the disruption of kinesin-1 (KIF5b) activity, an anterograde axonal motor protein related to microtubules and neurofilament. These findings indicate anterograde neurodegeneration in NMDA-induced neurotoxicity (Kuribayashi et al., 2010).

Glutamate was implicated to have a role in GON in an *in vivo* experimental glaucoma model (Kim et al., 2007, *Neuroscience*; Luo et al., 2009, *Cell. Mol. Neurobiol.*). IOP elevation causes stimulation of NMDA receptors in RGC bodies. Excessive levels of NMDA receptors cause apoptotic RGC death and subsequent axonal degeneration. Although the mechanism by which the stimulation of NMDA receptors occurs in high IOP-induced RGC death has not been fully elucidated, the inhibition of NMDA receptors may be important to prevent secondary degeneration in the optic nerve.

The elimination of excessive glutamate in the retina and vitreous is also important in slowing retinal neurodegenerative disease. The glutamate transporter is the only mechanism for the removal of glutamate from the extracellular fluid in the retina (Danbolt, 2001). Increased glutamate levels may result from a failure of glutamate transporters adjacent to RGCs. Mice deficient in the glutamate/aspartate transporter (GLAST) and excitatory amino acid carrier 1, which are glutamate transporters, demonstrate spontaneous RGC death, and optic nerve degeneration without IOP elevation, suggesting that glutamate transporters are necessary to prevent excitotoxic retinal damage (Harada et al., 1998, 2007; Sarthy et al., 2005). GLAST immunoreactivity is present throughout the retina and can be double-labeled with glutamine synthetase, a specific marker of Müller glial cells. These observations indicate that the intracellular glutamate concentration is dependent on glutamate uptake via GLAST in Müller glial cells.

### Endothelin in RGC death

Endothelin (ET)-1 is a well-known, potent, long-acting vasoconstrictor peptide in the CNS which reduces blood flow in the retina. There are two distinct G-protein-coupled receptors, the ET A receptor (ETAR) and ET B receptor (ETBR) that mediate the biological effects of ET. These receptors are abundantly expressed in various ocular tissues (MacCumber and D'Anna, 1994; Chauhan, 2008). ETAR is predominantly located in the retinal vessels and choroid vessels, and its activation sustains vasoconstriction of vascular smooth muscle cells. In contrast, ETBR is detected in neuronal cells and glial cells in the retina and optic nerve, where it causes the release of NO and results in transient vasodilation.

Activation of ET receptors was proposed as a mechanism of neuronal degeneration in the retina via ischemia and apoptotic cell death (Lau et al., 2006). Retrobulbar delivery of ET-1 can induce selective RGC degeneration, whereas intravitreal injection of ET-1 causes neuronal death not only of RGCs but also of other neurons. Since the mechanism by which ET-1 induces neuronal degeneration is mainly vasoconstriction, activated glia may also contribute. Our previous study showed that intravitreal injection of ET-1 induces phosphorylation of extracellular signal-regulated kinase in GFAP-positive glial cells. Furthermore, ET-1 induces matrix metalloproteinase (MMP)-2 and tissue inhibitors of matrix metalloproteinases (TIMPs)-1 and -2 as well as ECM protein expression, including fibronectin, in astrocytes (He et al., 2007). These observations implicate the relationship of MMP-2, TIMPs, and changes in the optic nerve head, including ECM remodeling, in ET-1-induced astrogliosis. In contrast, ET-1 increases intracellular NO, superoxide, and peroxynitrite in cultured retinal neurons. Furthermore, ET-1 causes increases in MMP-9 expression in RGCs and subsequently apoptotic RGC death (Aktas et al., 2010). Thus, ET signaling pathways exist not only in glial cells, such as astrocytes of the optic nerve head and Müller cells, but also in retinal neurons.

Several types of stress promote the activation of ET receptors in the optic nerve head and retina, with subsequent axonal degeneration and RGC death. Optic nerve crush promotes the expression of ETRA and ETRB in the optic nerve. Inhibition of ETRB by BQ-788 preserves RGCs, most likely by attenuating neuroinflammatory events, indicating RGC degeneration through the activation of ETRB (Tonari et al., 2012). Furthermore, IOP produces increased expression of ETRB in the retina, mainly RGCs; the nerve fiber layer (NFL); inner plexiform layer; and inner nuclear layer. RGC loss induced by high IOP is attenuated in ETRB-deficient (knockout) rats (Minton et al., 2012). These findings raise the possibility that ETRB contributes to glaucomatous RGC body death.

### RGC death in aging

Age is a well-recognized risk factor for the development of glaucoma. The prevalence of glaucoma in Caucasians in their 70 s can be 3.5–5-fold greater than that in their 40 s (Tielsch et al., 1991). However, the reason for this association with age has not been thoroughly elucidated. It is possible that older eyes are less resistant to elevated IOP. Age was found to be predictive of open-angle glaucoma development in patients with ocular hypertension (Gordon et al., 2002).

Many visual abilities decline with age as a result of the normal, non-pathologic loss of neurons in the peripheral and central visual pathways (Spear, 1993; Neufeld and Gachie, 2003). The age-related losses of neurons in the inner retina have been well documented. A decline in the number of neuronal cells is observed not only for RGCs, but also for interneurons in the INL and photoreceptors in the outer nuclear layer. The effect of aging has been investigated by both morphological analysis of RGC bodies and their axons in the optic nerve and by optical measurement of the retinal nerve fiber layer (RNFL) (Repka and Quigley, 1989; Harman et al., 2000). Investigations of the number of axons in the optic nerve found systemic age-related losses at rates ranging from 0.3 to 0.6% per year, whereas the age-related thinning of the RNFL occurs at a somewhat lower rate of 0.2% per year. The differences in age-related losses in RGCs and RNFL thickness suggest that axons do not represent a constant proportion of the total NFL thickness as shown by optical coherence tomography measurements. However, evidence for age-related variations in axon density in the RNFL requires quantitative modeling of RGCs and RNFL axons as a function of age.

Visual function also clearly declines with age. The majority of apparently disease-free individuals experience some degree of age-associated decline in vision (Spear, 1993). However, at least some age-related changes, including reductions in visual acuity, spatial contrast sensitivity, and motion sensitivity, cannot be attributed to optical changes. These defects are therefore likely to reflect changes in neurons, including those of the retina.

### Secondary degeneration of RGCs

The secondary degeneration of neighboring neurons is due to changes in surrounding biochemical events. A variety of mechanisms of secondary degeneration have been proposed, such as alteration of extracellular ion concentration, release of oxygen free radicals, and high levels of excitatory neurotransmitters (Liu and McAdoo, 1993; Dusart and Schwab, 1994; Faden, 1996). The experimental rat or mouse glaucoma model using IOP elevation may not be suitable for investigating secondary RGC degeneration, because rats and mice lack the lamina cribrosa, and therefore all RGCs axons are presumably exposed to the primary insult without banding of the lamina cribrosa (Morrison et al., 1995). In contrast, glaucoma patients exhibit pore banding in the lamina cribrosa, which results in partial axonal degeneration and fan-shaped RNFL thinning. RNFL thinning is commonly observed when glaucoma isrelatively advanced. At that time, there is still an opportunity to prevent further secondary RGC injury.

## Cell body protection

### Antioxidants

It has been suggested that antioxidant systems, which remove free radicals and inhibit the production and release of ROS, are activated when superoxide and NO are abundantly observed in cells. A decrease in free radicals protects cellular and subcellular integrity under hypoxic conditions. In addition, suppression of free radical-mediated lipid peroxidation directly interferes with the early phase of apoptotic cell death. The lipid peroxidation inhibitor tirilazad mesylate partially prevents RGC death induced by the absence of neurotrophins in mixed culture. Furthermore, the flavonoids baicalin and genistein also attenuate rotetone-induced oxidative stress through the inhibition of lipid peroxidation (Kamalden et al., 2012).

Overexpression of redox proteins, such as Trx1 and Trx2, in RGC-5 cells contributes to cell survival against glutamate/BSO-induced cytotoxicity (Munemasa et al., 2008). It has been assumed that Trx1 inhibits the downstream release of ASK1 from the Trx1/ASK1 complex in the cytosol, resulting in the inhibition of the JNK and p38 apoptotic pathway (Nadeau et al., 2007). Trx2 regulates mitochondria without membrane permeabilization and apoptosis via a redox-active site cysteine-independent mechanism, including inhibition of release of cytochrome C (Wang et al., 2006). Modulation of the redox status may therefore become an additional strategy for cytoprotection against apoptotic RGC body death.

### Gene therapy

Electroporation can introduce large molecules such as DNA into cells. Recently, we have modified a previous method and succeeded in gene delivery to RGCs with greater than 40% transfection efficiency (Munemasa et al., 2008). Overexpression of Trx1 and Trx2 with electroporation after intravitreal injection of these plasmid DNAs contribute to cell survival in ocular hypertension and after optic nerve transection-induced RGC body death. In addition, Trx2 overexpression with electroporation was confirmed using immunoblotting not only in the retina but also in the optic nerve, indicating successful gene delivery to the optic nerve. Our recent work has shown that overxpression of Nell2, a neuron-specific thrombospondin-1-like extracellular protein, also preserves axotomized RGCs through MACF1 interaction (Munemasa et al., 2012). Although gene delivery to RGCs by electroporation can reduce the impact of various forms of stress, the clinical application of this strategy is limited by the efficiency of transfection.

Adenoviral vectors are effective in reducing functional damage in several models of ocular disease (Ali et al., 2000; Acland et al., 2001). Viruses were selected during evolution to enter cells cleanly and efficiently. BDNF-expressing modified adeno-associated virus (AAV) temporarily delays RGC degeneration after IOP elevation, with long-term transgene effects (Martin et al., 2002). A recent study has shown that the intramuscular injection of systemic AAV carrying erythropoietin (rAAV2/5. CMV.EpoR76) protects against RGC body death and axonal degeneration in DBA/2J glaucomatous mice. In addition, the systemically administered mutated gene can reduce the toxic side effects of AAV (Sullivan et al., 2011). Thus, inserting a neuroprotective candidate into AAV and systemic administration or intravitreal injection may become a potential adjunctive therapy to prevent RGC body death.

## Axonal injury

### Laminar and BDNF

It has been suggested that oligodendrocyte loss leads to a loss of axons in a hypertensive glaucoma model (Nakazawa et al., 2006). On the other hand, astrocytes play crucial roles in the early stage of axon loss, whereas oligodendrocyte loss occurs after axons have already degenerated in hypertensive glaucoma (Son et al., 2010). Among oligodendrocytes, astrocytes, and microglia, the loss of BDNF may specifically affect the oligodendrocyte lineage cell population, and BDNF may be a critical factor in the development of oligodendrocytes and the recovery of oligodendrocytes from a demyelinating lesion (VonDran et al., 2011). It was shown that the axon diameter and proportion of myelinated axons were reduced in the optic nerve in mice lacking BDNF (Cellerino et al., 1997). An increase in astrocyte number was observed in the optic nerve in the mouse glaucoma model, indicating that astrocytes were activated, thereby leading to demyelination in association with the process of axon loss (Mabuchi et al., 2003). On the other hand, previous studies demonstrated that the activation of microglia contributes to axon damage in glaucoma (Yuan and Neufeld, 2001). Optic nerve degeneration was paralleled by a loss of axons and an increase in microglia in the DBA/2NNia mouse (May and Mittag, 2006). However, to the best of our kowledge, there is no report showing that the activation of oligodendrocytes leads to axonal damage in glaucoma. These findings have been implicated that the activation of astrocytes and microglia and decrease in oligodendrocytes may be associated with axonal damage. Endogenous BDNF is present predominantly in myelin rather than in the axoplasm in the myelinated optic nerve axon (Fujino et al., 2009). Previous studies demonstrated that endogenous BDNF is transported from synapses to RGC bodies, thereby contributing to RGC survival (DiStefano et al., 1992; Quigley et al., 2000). Obstruction of BDNF axonal transport has been reported in the unmyelinated optic nerve in a hypertensive glaucoma model (Pease et al., 2000). In the normal spinal cord, most oligodendrocytes exhibit BDNF immunoreactivity (Dougherty et al., 2000). In the basal forebrain, oligodendrocytes were shown to provide BDNF to nearby neurons (Dai et al., 2003). Release of BDNF from oligodendrocytes is regulated through metabotrophic glutamate receptors, and cortical oligodendrocytes also provide trophic support to neurons (Bagayogo and Dreyfus, 2009). Myelin containing BDNF terminates at the laminar portion between the myelinated and unmyelinated areas in the optic nerve. This may be involved in the disruption of BDNF axonal transport at the laminar portion under certain conditions. Thus, not only mechanical obstruction, such the relationship between myelin and axons at the laminar portion, may be one causative factor of glaucomatous damage starting at the laminar portion.

### Mitochondria and axonal degeneration

Optic atrophy type 1 (Opa1) is a component of the mitochondrial network and plays a role in the regulation of mitochondria in RGC pathophysiology (Delettre et al., 2000). Although Opa1 does not promote mitochondrial docking, it may affect the fusion step to regulate mitochondrial morphology (Cipolat et al., 2004). A recent study has demonstrated an increased synaptic vesicle number in bipolar cell terminal arbors without significant loss of mitochondrial membrane potential in the retina and optic nerve, suggesting that Opa1 may be an essential factor in RGC synaptic architecture and connectivity (Williams et al., 2012). Compensation of oxidative phosphorylation function may be important for maintaining ATP synthesis in early stage OPA1-related optic neuropathy (Van Bergen et al., 2011). A recent meta-analysis study has shown an association between OPA1 polymorphisms and the risk for normal tension glaucoma in Caucasians but not in Asians (Guo et al., 2012). That study also found no difference in the association between OPA1 polymorphisms and hypertensive glaucoma in Asians and Caucasians (Guo et al., 2012). An investigation using human lamina cribrosa cells study which obtained from donors with no history of glaucoma and from donors with glaucoma demonstrated increased ROS production, compromised antioxidant capacity, mitochondrial dysfunction, and dysfunctional Ca^2+^ homeostasis in glaucomatous lamina cribrosa cells compared with normal lamina cribrosa cells and found that the capacity of glaucomatous lamina cribrosa cells to inhibit the increase in ROS production appeared to be impaired (McElnea et al., 2011). Therefore, mitochondrial dysfunction in lamina cribrosa cells may be involved in glaucoma pathophysiology (McElnea et al., 2011). OPA1 expression and the OPA1/β-globulin ratio were both significantly lower in primary open-angle glaucoma (POAG) patients than in controls, suggesting that decreased OPA1 expression in those patients may contribute to RGC apoptosis as one primary mechanism of optic nerve damage (Bosley et al., 2011). The concentration of mitochondria in unmyelinated axons is much higher than that in myelinated axons in the optic nerve (Perge et al., 2009). An increase in mitochondrial OPA1 has been suggested to be an important cellular defense mechanism against hypertensive-mediated RGC damage (Dai et al., 2011), and such an endogenous defense response can be seen in the cell bodies. In addition, mitochondrial matrix swelling and cristae volume depletion were reported in the unmyelinated optic nerve head of glaucomatous DBA/2J mice (Ju et al., 2008). The number of Mitotracker-labeled mitochondria decreased in the unmyelinated optic nerve portion in a hypertensive glaucoma model (Munemasa et al., 2010). Thus, not only mitochondrial dysfunction in lamina cribrosa cells but also in the axons at the laminar portion may also be one causative factor of glaucomatous optic nerve damage.

### TNF and axonal degeneration

TNF has been linked to optic nerve degeneration in glaucoma patients (Yan et al., 2000; Yuan and Neufeld, 2000; Tezel et al., 2001). TNF release by glial cells was induced on exposure to simulated ischemia and elevated hydrostatic pressure, resulting in apoptotic RGC death (Tezel and Wax, 2000). A recent study has shown that a significantly higher percentage of patients in the glaucoma group were positive for TNF in the aqueous humor compared with the cataract group (Sawada et al., 2010). More recently, a study of proteomic data from human glaucoma has shown a prominent upregulation of TNF/TNF receptor 1 signaling in the glaucomatous retina (Yang et al., 2011). It was reported that a significant elevation of endogenous TNF occurs in the retina in a hypertensive glaucoma mouse model (Nakazawa et al., 2006). In addition, a significant increase in TNF levels was observed in the optic nerve in a hypertensive glaucoma rat model (Munemasa et al., 2010). Exogenous TNF was shown to cause primary optic nerve axonal degeneration with subsequent slow RGC body death (Kitaoka et al., 2006; Nakazawa et al., 2006). Microglial activation and upregulation of nuclear factor-κ B p65 may be involved in this process (Kitaoka et al., 2006). Taken together, the results indicate that TNF released from glial cells may play a pivotal role in axonal degeneration in glaucoma. This finding is supported by a more recent study showing that etanercept, a TNF blocker, exerted axonal protection in a rat hypertensive glaucoma model (Roh et al., 2012).

## Axonal protection

### BDNF and axonal protection

A recent study has demonstrated that transplantation of BDNF-secreting mesenchymal stem cells exerts optic nerve protection in a hypertensive glaucoma rat model (Harper et al., 2011). It was reported that intravitreal injection of BDNF significantly prevented axonal loss after optic nerve crush in cats, and the injection combined with delivery of BDNF to the visual cortex exerted greater protection (Weber et al., 2010). BDNF can induce phosphorylation of the cAMP response element-binding (CREB) protein in certain neurons (Finkbeiner et al., 1997; Watson et al., 1999). Several reports suggested a prosurvival role of the CREB protein in the CNS (Walton et al., 1999; Lonze and Ginty, 2002). The expression of constitutively active CREB protein is sufficient to promote regeneration of dorsal root ganglion axons (Gao et al., 2004). Since CREB-binding sequences have been identified in the BDNF gene (Tao et al., 1998), BDNF can be upregulated by CREB (Bonni et al., 1999; Freeland et al., 2001), and therefore the CREB may be located both upstream and downstream of BDNF. We previously demonstrated that intravitreal injection of BDNF can upregulate the p-CREB protein level in nuclei in oligodendrocytes, with subsequent regulation of BDNF expression in the optic nerve, leading to axonal protection against TNF-induced optic nerve degeneration (Fujino et al., 2009). Since it was demonstrated that BDNF is produced by oligodendrocytes and can provide local trophic support for nearby neurons in the basal forebrain (Dai et al., 2003), it is possible that BDNF in oligodendrocytes may serve as a protective moderator for optic nerve axons. It is interesting to note that brimonidine, which can potentially upregulate BDNF (Gao et al., 2002), preserved optic nerve axons in a hypertensive rat glaucoma model (Lambert et al., 2011). Moreover, the compound *bis*(3-propionic acid methyl ester)phenylphosphine borane reducing complex 1 exhibits axonal protection in the optic nerve in a hypertensive glaucoma rat model with increasing BDNF levels (Almasieh et al., 2011). Thus, BDNF may have beneficial effects on not only on RGC bodies but also on their axons in glaucoma.

### Nicotinamide adenine dinucleotide and axonal protection

Nicotinamide adenine dinucleotide (NAD), a key intermediate in cellular energy homeostasis, has been suggested to play crucial roles in axonal degeneration in cultured neurons. SIRT1 is a member of a highly conserved gene family (sirtuins) encoding NAD^+^-dependent deacetylases (North and Verdin, 2004). SIRT1 activation or exogenous NAD supply may prevent axonal degradation in dorsal root ganglia explant cultures (Araki et al., 2004). Previous studies demonstrated a decline in NAD levels in the transected wallerian degenerating axons of dorsal root ganglia (Wang et al., 2005) and a decline in NAD levels in the cervical spinal cords of an experimental autoimmune encephalomyelitis model (Kaneko et al., 2006), suggesting that decreased NAD may be involved in axonal degeneration. Our previous study also showed a decrease in NAD levels in the optic nerve in a TNF-induced axonal degeneration model and that the exogenous NAD supply can exert axonal protection in the optic nerve (Kitaoka et al., 2009). In addition, axonal density in optic nerves, 14 and 30 days postimmunization demonstrated that the administration of oral SRT501, a SIRT1 activator, prevented the loss of axons found in experimental autoimmune encephalomyelitis-related optic neuritis eyes (Shindler et al., 2010). Nicotinamide monucleotide is directly synthesized into NAD by nicotinamide mononucleotide adenylyltransferase (Nmnat), an essential enzyme that catalyzes the final step of NAD biosynthesis. Several studies demonstrated protective roles of Nmnat isoforms in axonal degeneration. Nmnat3, located in mitochondria, can prevent axonal degeneration triggered by ROS in dorsal root ganglia culture (Press and Milbrandt, 2008). It was shown that exogenous Nmnat2, located in the cytosol, only confers significant protection on cut neurites when expressed at high levels and endogenous Nmnat2 is essential for the maintenance of healthy axons (Gilley and Coleman, 2010). Although the protective role of Nmnat1 remains to be elucidated (Araki et al., 2004; Conforti et al., 2007), it may confer axonal protection when it is present not only in the nucleus but also in the cytoplasm (Yahata et al., 2009). It is possible that Nmnat1 plays a substantial role in optic nerve axonal degeneration since we observed that Nmnat1 is present in optic nerve axons and RGC bodies, and a decline in Nmnat1 precedes axonal degeneration (Kitaoka et al., 2009). However, this finding may be dependent on the type of injury or on the neuronal cell type because endogenous Nmnat1 does not have a primary role in axon maintenance in sciatic nerves and superior cervical ganglia (Conforti et al., 2011).

### Trx and axonal protection

**Figure 1:**
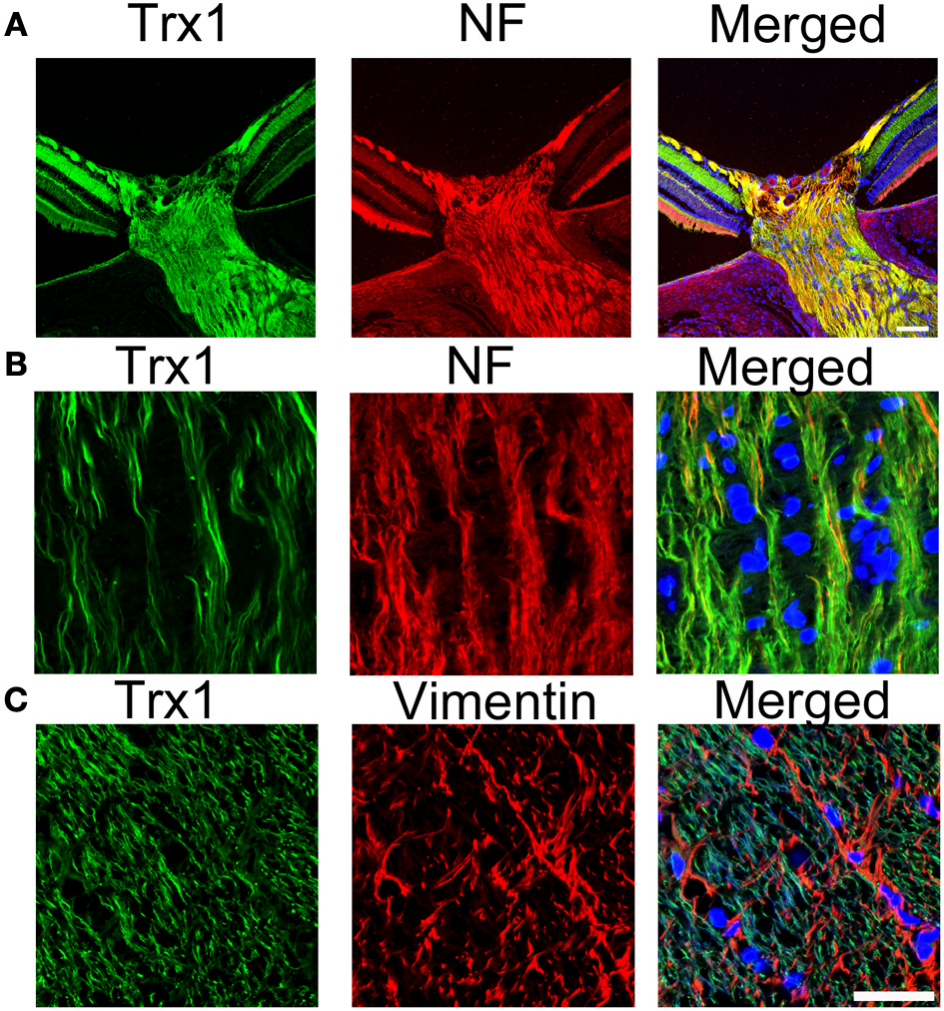
**Localization of Trx1 in the optic nerve. (A)** and **(B)** Double staining for Trx1 (green) and neurofilament (red). Substantial co-localization of Trx1 and NF is observed in the PBS-treated optic nerve. **(C)** Double staining for trx1 (green) and vimentin (red). No colocalization of Trx1 and Vimentin is observed in the PBS-treated optic nerve. Scale bar, 25 μm. Trx1, trioredoxin1; NF, neurofilament.

Trx has two isoforms, Trx1 and Trx2, located in the cytosol and mitochondria, respectively. Trx1 can be transported anterogradely by axoplasmic transport in the sciatic nerve (Stemme et al., 1985). It was suggested that Trx1 is required for the neurite outgrowth of PC12 cells induced by nerve growth factor (Bai et al., 2003). In addition, it was suggested that the continued synthesis of Trx1 protein by nerve cells themselves and the secretion of Trx1 from nerve cells may help to protect cells via an antioxidative role (Lippoldt et al., 1995). In the optic nerve, Trx1 is present in the axoplasm and colocalized with neurofilament in both the unmyelinated and myelinated areas (Figures 1A and B). Trx1 is not colocalized with vimentin-positive astrocytes (Figure 1C). Our previous study demonstrated that a decrease in Trx1 protein levels in the optic nerve may precede axonal loss and that Trx1 induction may participate in 17β-estradiol-mediated axonal protection (Kitaoka et al., 2011). It was reported that 17β-estradiol increased the expression of Trx1 through the cGMP and protein kinase G signaling pathway in neuronal cells (Lee et al., 2003). In addition, Trx1 synthesis may be involved in axonal regeneration, and its inhibition is associated with reduced neurite outgrowth of dorsal root ganglia neurons (Tonge et al., 2008). Both Trx1 and Trx2 may participate in axonal protection. In a hypertensive glaucoma rat model, a significant decrease in Trx2 levels was observed in the mitochondrial fraction of the optic nerve compared with control optic nerves (Munemasa et al., 2010). AIF is a phylogenetically conserved redox-active flavoprotein that contributes to cell death and oxidative phosphorylation (Susin et al., 1999). AIF is activated by its translocation to the cytoplasm and nucleus under various stresses due to an increase in mitochondrial membrane permeability. The inhibition of AIF translocation by Trx2 overexpression has cytoprotective effects against oxidative stress-induced neurotoxicity by decreasing the mitochondrial membrane permeability. Our previous data showed that increased Trx2 in the optic nerve inhibited AIF translocation from the mitochondria to axoplasm, thereby leading to axonal protection against optic nerve degeneration in a hypertensive glaucoma model (Munemasa et al., 2010). Although apoptosis may occur in RGC bodies, local axonal AIF translocation from the mitochondria to axoplasm may play a crucial role in axonal degeneration in glaucoma.

## Future directions

**Figure 2:**
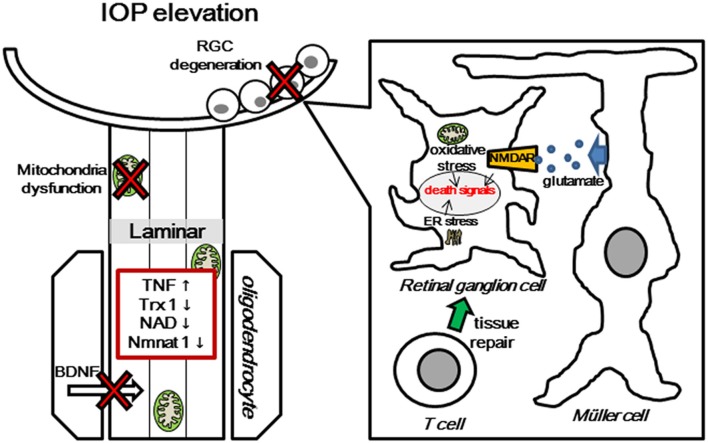
**Multiple pathogenic and biological mechanisms for glaucomatous neurodegeneration.** Increased IOP induces several molecular changes, such as glial activation and release of TNF, mitochondrial dysfunction, degradation of Trx1, NAD, and nmnat1, and obstruction of BDNF transport at lamina area. Molecular changes in the axon cause death signal activation in RGC body, including release of ROS from mitochondria, autoimmune dysregulation, and avtivation of glutamate transporter. TNF, Tumor Necrosis Factor; Trx1, thioredoxin1; NAD, nicotinamide adenine dinucleotide; BDNF, Brain-derived neurotrophic factor; NMDAR, N-methyl-D-aspartate receptor; ER, endoplasmic reticulum.

The molecular mechanism of RGC death has been investigated in several laboratories. However, the molecular mechanism of local axonal degeneration in glaucoma has not been elucidated thoroughly. Our laboratory is addressing this mechanism, and its clarification may be important for developing neuroprotective agents (Figure 2). Recent imaging techniques allow us to see RGC bodies and the NFL in glaucoma patients. A very recent study has demonstrated that adaptive optic scanning laser ophthalmoscopy can visualize and assess the surface-level pores of the lamina cribrosa in patients with glaucoma (Akagi et al., 2012). Therefore, it will feasible to examine individual axons and cell bodies in glaucoma patients in the near future. In addition to the reduction of IOP, the concept of axonal protection may become a new therapeutic strategy to prevent further axonal degeneration or revive dying axons in patients with preperimetric glaucoma. Further study will be needed to clarify whether the combination therapy of axonal protection and cell body protection will have greater protective effects in early or progressive GON.

### Conflict of interest statement

The authors declare that the research was conducted in the absence of any commercial or financial relationships that could be construed as a potential conflict of interest.
